# Serum insulin-like growth factor-1 and its association with epilepsy risk and cognitive function

**DOI:** 10.1186/s42494-026-00274-2

**Published:** 2026-10-01

**Authors:** Jiahui Guo, Zhiruo Qiu, Bofei Chen, Haotian Li, Ping Su, Zhefeng Yuan, Dan Wu, Jiajia Fang

**Affiliations:** 1 Department of Neurology, The Fourth Affiliated Hospital of School of Medicine and International School of Medicine, International Institutes of Medicine, Zhejiang University, Yiwu, 322000 China; 2 College of Biomedical Engineering & Instrument Science, Zhejiang University, Hangzhou, Zhejiang 310027 China; 3 Department of Neurology, Center for Membrane Receptor and Brain Medicine, The Fourth Affiliated Hospital of School of Medicine and International School of Medicine, International Institutes of Medicine, Zhejiang University, Yiwu, 322000 China; 4 Department of Neurology, Children’s Hospital, Zhejiang University School of Medicine, National Clinical Research Center for Child Health, Hangzhou, 310003 China; 5 The Fourth Affiliated Hospital, School of Medicine, Zhejiang University, N1 Shangcheng Avenue (North), Yiwu City, Zhejiang Province China; 6 N38 Zheda Road, Xihu District, Hangzhou City, Zhejiang Province China

**Keywords:** Epilepsy, Insulin-like growth factor 1, Cognition, Biomarker, Susceptibility

## Abstract

**Background:**

Insulin-like growth factor 1 (IGF-1) has neurotrophic and neuroprotective properties, but its relationship with epilepsy risk and cognitive outcomes remains unclear. We investigated whether serum IGF-1 was associated with incident epilepsy and cognitive performance in a large population-based cohort.

**Methods:**

We performed a prospective analysis of the UK Biobank including 466,857 participants with baseline serum IGF-1 measurements. Incident epilepsy was identified from hospital records and self-report. Associations with epilepsy risk were assessed using prespecified Cox proportional hazards models, restricted cubic splines, and sensitivity analyses. Cross-sectional and longitudinal analyses were used to examine cognitive outcomes.

**Results:**

Over more than 15 years of follow-up, 4,532 participants developed incident epilepsy. Higher serum IGF-1 was consistently associated with a lower risk of incident epilepsy; in the primary model, each 1 nmol/L increase was associated with a lower hazard of incident epilepsy (HR = 0.978, 95% CI 0.973–0.984). The association was nonlinear and L-shaped, with a steeper increase in risk at lower IGF-1 concentrations and an apparent plateau above approximately 20 nmol/L. Per-standard-deviation, quartile-based, lagged, and alcohol-frequency sensitivity analyses showed similar results. In cross-sectional analyses of participants with epilepsy at baseline, higher IGF-1 was nominally associated with faster reaction time and better prospective memory, but neither association remained significant after false discovery rate correction. Exploratory longitudinal analyses in the epilepsy subgroup were limited by sample size and did not show significant cross-lagged associations. In the full cohort, higher baseline IGF-1 was associated with better subsequent performance in several cognitive domains, although these findings were not epilepsy-specific.

**Conclusions:**

Lower serum IGF-1 was independently associated with a higher risk of incident epilepsy in this large population-based cohort. Evidence for cognitive associations was more limited, with only nominal cross-sectional findings in baseline epilepsy and exploratory, non-epilepsy-specific longitudinal signals. These results support further investigation of circulating IGF-1 as a candidate biomarker and biologically relevant pathway rather than as evidence of a causal or therapeutic target.

**Graphical Abstract:**

**Figure:**
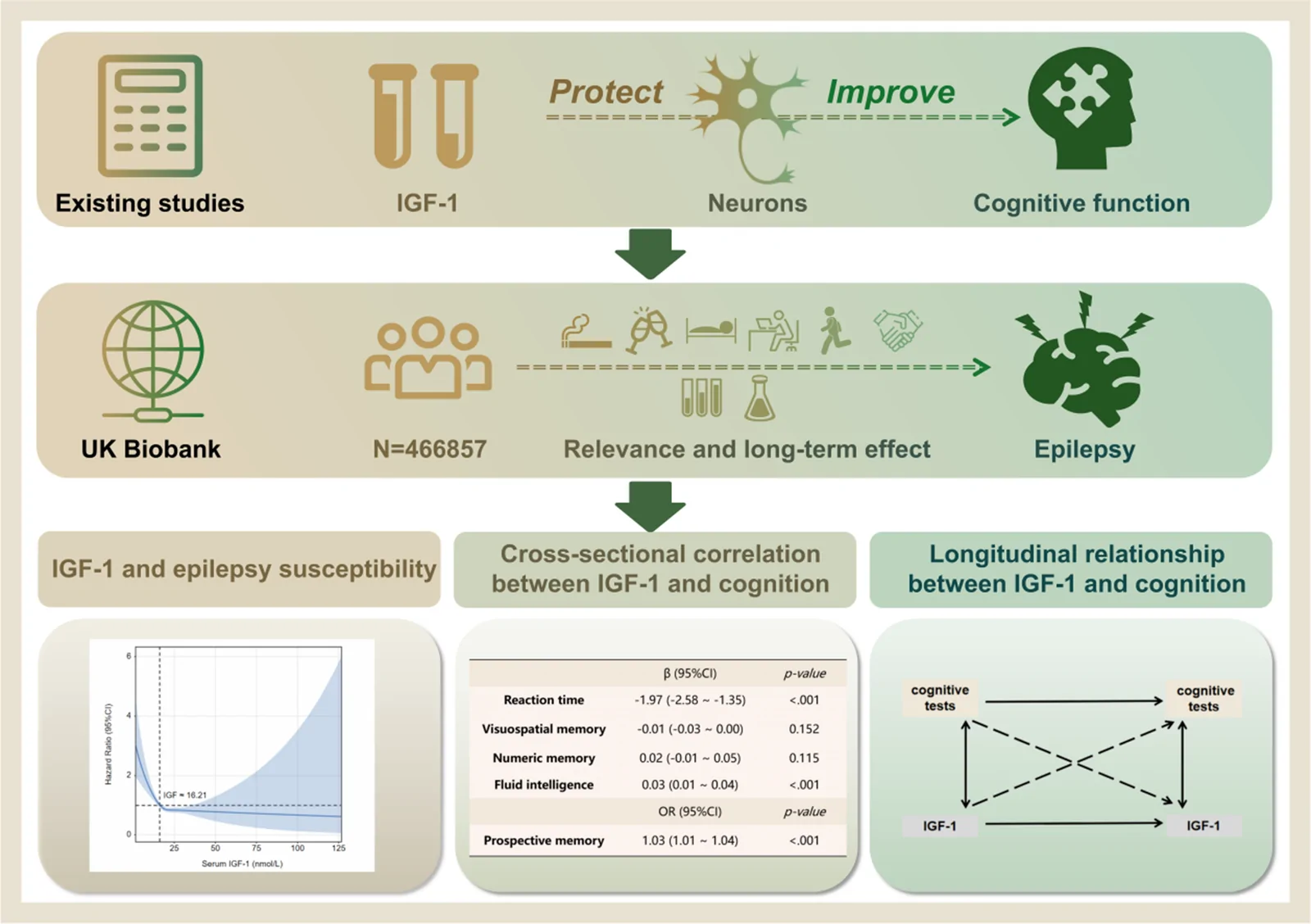

**Supplementary Information:**

The online version contains supplementary material available at https://doi.org/10.1186/s42494-026-00274-2.

## Background

Epilepsy is a common and heterogeneous neurological disorder characterized by an enduring predisposition to generate seizures and by a broad spectrum of neurobiological, cognitive, and psychosocial consequences [1–3]. Beyond seizure occurrence itself, cognitive impairment is among its most clinically relevant comorbidities [4, 5], affecting attention, processing speed, memory, and executive function, and thereby contributing substantially to reduced educational attainment, impaired employment, and diminished quality of life [6–9]. At the same time, there is growing recognition that cognitive and behavioral abnormalities may precede, accompany, or follow epilepsy, underscoring the need for biomarkers that capture disease risk and brain health more broadly rather than seizure burden alone [10–12].

Insulin-like growth factor 1 (IGF-1) is a pleiotropic peptide hormone, produced predominantly in the liver under growth hormone regulation but also expressed in extrahepatic tissues, including the brain. IGF-1 signaling has been implicated in neurodevelopment, neuronal survival, synaptic plasticity, myelination, and neuroimmune regulation [13–18], making it biologically plausible as a marker of vulnerability or resilience in neurological disease [19–21]. Recent work has further strengthened this framework by showing that microglia regulate prenatal human GABAergic neurogenesis through IGF-1 signaling, highlighting a potential role for this pathway in shaping inhibitory circuit development at an early stage [22]. These observations are relevant to epilepsy, in which altered inhibitory–excitatory balance, network maturation, and neuroinflammatory signaling are central pathophysiological themes.

In epilepsy specifically, however, the translational significance of IGF-1 remains uncertain. Experimental studies suggest that disrupted IGF-1 signaling may influence seizure generation or epileptic network development in selected models [22–25], yet clinically established circulating biomarkers for predicting incident epilepsy or tracking epilepsy-related cognitive burden remain lacking [10, 26–29]. Moreover, although lower circulating IGF-1 has been linked to less favorable brain health and cognition in large population datasets, its relationship to incident epilepsy at the population level has not been clearly defined [30]. Clarifying this association is important, not because serum IGF-1 can currently be considered a diagnostic or therapeutic tool in epilepsy, but because it may provide epidemiological evidence relevant to risk stratification and hypothesis generation.

Against this background, we examined whether circulating IGF-1 was associated with incident epilepsy and cognitive measures in the UK Biobank. Specifically, we investigated the relationship between baseline serum IGF-1 concentrations and subsequent epilepsy risk in participants free of epilepsy at enrollment, and we further explored whether serum IGF-1 was related to cognitive performance in epilepsy and in the cohort more broadly. We hypothesized that lower circulating IGF-1 would be associated with a higher risk of incident epilepsy and with less favorable cognitive phenotypes, while interpreting these findings as observational associations rather than evidence of causality or therapeutic effect.

## Methods

### Study design and population

This prospective cohort study was conducted within the UK Biobank, a large population-based resource that recruited more than 500,000 participants aged 40–69 years between 2006 and 2010 across 22 assessment centres in England, Scotland, and Wales [31, 32]. Participants completed baseline questionnaires, physical assessments, and biospecimen collection, with subsequent linkage to national health records. For the present study, we included participants with available baseline serum IGF-1 measurements. The study was conducted in accordance with the Strengthening the Reporting of Observational Studies in Epidemiology (STROBE) statement [33]. All participants provided written informed consent, and UK Biobank has ethical approval from the North West Multi-Centre Research Ethics Committee.

### Measurement of IGF-1

Serum IGF-1 concentrations were measured from blood samples collected at recruitment. Sample processing, handling, and long-term storage followed the standardized UK Biobank protocol, which was designed to preserve biospecimen integrity for large-scale epidemiological analyses [34]. Serum IGF-1 was assayed using a centralized chemiluminescent immunoassay platform. External quality assurance procedures were also applied to support analytical reliability.

### Cognitive function tests

Cognitive performance was assessed using UK Biobank touchscreen-based cognitive tasks at baseline, with selected domains reassessed during follow-up. The psychometric characteristics, concurrent validity, and short-term test–retest reliability of the UK Biobank cognitive tests have been described previously [35, 36]. Five cognitive domains were analyzed in the present study: visuospatial memory, prospective memory, fluid intelligence, numeric memory, and reaction time. Visuospatial memory was defined as the number of incorrect symbol matches after a brief visual presentation; higher values indicate worse performance. Prospective memory assessed whether the participant correctly executed a previously instructed action after a delay/distraction period. Fluid intelligence was defined as the number of verbal and numerical reasoning items answered correctly within 2 min. Numeric memory was defined as the maximum digit span recalled correctly. Reaction time was defined as the response latency in a card-matching task; higher values indicate worse performance.

### Ascertainment of epilepsy

The primary outcome was incident epilepsy during follow-up. UK Biobank participants are linked to routinely collected electronic health records through the National Health Service. Epilepsy diagnoses were identified using the Tenth Revision of the International Classification of Diseases (ICD-10), specifically codes G40–G41. The operational definition of epilepsy was aligned with the contemporary clinical framework proposed by the International League Against Epilepsy [37]. Participants with epilepsy at baseline were identified separately for cross-sectional cognitive analyses. For participants free of epilepsy at baseline, follow-up accrued from recruitment until incident epilepsy, death, loss to follow-up, or October 30, 2023, whichever came first.

### Assessment of covariates

Potential confounders were selected a priori on clinical and epidemiological grounds. These included age, sex, ethnicity, education, Index of Multiple Deprivation (IMD), body mass index (BMI), smoking, alcohol use, sleep hours per day, physical activity minutes per week, working hours per week, and loneliness. In the primary analyses, alcohol use was coded as a binary variable. In additional sensitivity analyses, alcohol use was re-coded using the available baseline UK Biobank drinking-frequency categories. Participants were classified as never drinkers, occasional drinkers, regular weekly drinkers, or daily/almost daily drinkers. Responses of “prefer not to answer” and missing values were treated as missing.

### Statistical analysis

Descriptive statistics were used to summarize baseline characteristics. Categorical variables are presented as number (percentage), and continuous variables as mean ± standard deviation. For baseline comparisons across the three epilepsy-status groups, categorical variables were compared using the χ² test. Continuous variables were compared using one-way analysis of variance (ANOVA) when distributional assumptions were approximately satisfied and using the Kruskal–Wallis test otherwise. Post hoc pairwise comparisons were performed only when the overall test was statistically significant. Tukey’s honestly significant difference test was used after ANOVA, whereas Dunn’s test with Bonferroni correction was applied after Kruskal–Wallis testing.

Among participants free of epilepsy at baseline, Kaplan–Meier curves were used to compare epilepsy-free survival according to baseline serum IGF-1 categories, and differences were assessed with the log-rank test. Cox proportional hazards regression models were then used to examine the association between serum IGF-1 and incident epilepsy, with results reported as hazard ratios (HRs), 95% confidence intervals (CIs), and two-sided *P* values. Covariates were prespecified rather than selected on the basis of univariable significance, consistent with recommended regression modeling principles [38]. The primary model was adjusted for age, sex, BMI, smoking, alcohol use, sleep hours per day, and loneliness. The sensitivity model additionally included ethnicity, education, deprivation, physical activity, and working hours in a reduced complete-case sample. Follow-up time was modeled in days from baseline to incident epilepsy or censoring. Missing data were handled using complete-case analysis, and analytic sample sizes therefore differed across models.

To improve interpretability, serum IGF-1 was analyzed in several complementary ways. First, it was modeled as a continuous variable per 1 nmol/L increase. Second, it was standardized and analyzed per 1 standard deviation (SD) increase. Third, participants were categorized into quartiles according to the baseline serum IGF-1 distribution, with the lowest quartile used as the reference group. For quartile-based analyses, event counts, person-years, incidence rates per 1,000 person-years, and estimated 15-year cumulative incidence were calculated. A *P*-value for trend across quartiles was also estimated.

To reduce concern about reverse causation, lagged Cox analyses were performed excluding epilepsy events occurring within the first 2 and 5 years of follow-up. For each lag analysis, only participants who remained under observation and free of epilepsy beyond the corresponding landmark time were included, and follow-up time was redefined from that landmark onward. The same covariate structure as in the main Cox analyses was retained.

Because the inverse association between alcohol use and incident epilepsy was potentially counterintuitive, additional sensitivity analyses replaced the binary alcohol variable with the refined alcohol-frequency variable described above. Cox models were re-fitted using the same covariate structure as in the main analyses. A further sensitivity analysis excluded participants who reported drinking daily or almost daily.

The dose–response relationship between serum IGF-1 and incident epilepsy was further examined using restricted cubic splines within the Cox framework. Restricted cubic splines are widely used to flexibly model non-linear exposure–outcome associations in epidemiological data [39]. Five knots were placed at the 5th, 35th, 50th, 65th, and 95th percentiles of the serum IGF-1 distribution.

For cognitive analyses, univariable and multivariable regression models were used to examine the association between serum IGF-1 and performance across five cognitive domains in participants with epilepsy at baseline. Linear regression was used for continuous cognitive outcomes, and ordinal logistic regression was used for prospective memory. To account for multiple testing across the five cognitive domains, *P*-values for the serum IGF-1 term from the fully adjusted model were additionally corrected using the Benjamini–Hochberg false discovery rate (FDR) procedure. Longitudinal associations between serum IGF-1 and cognitive performance were explored using cross-lagged panel models, which are commonly used to examine directional associations between repeatedly measured variables over time, while acknowledging the interpretive limitations of this framework [40]. All analyses were performed in R version 4.3.2. Two-sided *P*-values are reported, and statistical significance was defined as *P* < 0.05; where FDR correction was applied, adjusted *q* values are additionally reported.

### Data availability

The data that support the findings of this study are available from the UK Biobank resource. Access to these data is subject to approval by the UK Biobank Access Management System and is therefore not publicly available. Researchers may apply for access directly through the UK Biobank website. The authors do not have permission to make the data publicly available.

### Ethics

UK Biobank data use for the present study (application no. 91493) was approved according to UK Biobank access procedures. UK Biobank has approval from the North West Multi-Centre Research Ethics Committee as a Research Tissue Bank, which permits approved researchers to conduct analyses under that overarching ethical framework. All participants provided informed consent at recruitment.

## Results

### Baseline characteristics

A total of 466,857 participants were included in the study, comprising 2,225 individuals with epilepsy at baseline, 4,532 who developed epilepsy during follow-up (incident epilepsy), and 460,100 who remained free of epilepsy throughout follow-up. Baseline characteristics stratified by epilepsy status are shown in Table 1. Across the three groups, significant overall differences were observed for age, sex, education, BMI, IMD, physical activity minutes per week, working hours per week, smoking, alcohol, loneliness, serum IGF-1, visuospatial memory, prospective memory, numeric memory, reaction time, and fluid intelligence (all *P* overall < 0.05). In contrast, ethnicity did not differ significantly across groups (*P* overall = 0.163), and sleep hours per day was borderline and did not meet the predefined significance threshold (*P* overall = 0.050).

**Table 1:** Baseline characteristics of the study population stratified by epilepsy status

	Total (*n* = 466857)	Epilepsy at baseline (*n* = 2225)	Epilepsy during follow-up (*n* = 4532)	No epilepsy all the time (*n* = 460100)	*P* overall
**Sex**					< 0.001
Female	253,102 (54.2%)	1067 (48.0%)	2189 (48.3%)	249,846 (54.3%)	
Male	213,755 (45.8%)	1158 (52.0%)	2343 (51.7%)	210,254 (45.7%)	
**Age**,** years**	56.53 ± 8.09	56.37 ± 8.13	58.41 ± 7.92	56.52 ± 8.09	< 0.001
**Ethnicity**					0.163
White	409,022 (94.7%)	1963 (95.3%)	3985 (95.2%)	403,074 (94.7%)	
Other	22,847 (5.3%)	97 (4.7%)	200 (4.8%)	22,550 (5.3%)	
**Education**					< 0.001
College or University degree	151,176 (39.6%)	488 (31.8%)	1166 (35.2%)	149,522 (39.7%)	
Other qualifications	230,515 (60.4%)	1046 (68.2%)	2142 (64.8%)	227,327 (60.3%)	
**BMI**,** kg/m²**	27.42 ± 4.78	28.20 ± 5.48	27.99 ± 5.09	27.41 ± 4.77	< 0.001
**IMD**	17.59 ± 13.94	23.13 ± 16.64	20.11 ± 16.05	17.54 ± 13.90	< 0.001
**Physical activity minutes per week**	936.06 ± 1227.87	944.82 ± 1289.63	950.88 ± 1262.17	935.88 ± 1227.26	< 0.001
**Sleep hours per day**	7.10 ± 1.29	7.07 ± 2.00	7.07 ± 1.67	7.10 ± 1.29	0.050
**Working hours per week**	34.74 ± 13.41	33.04 ± 13.42	33.81 ± 13.62	34.75 ± 13.41	< 0.001
**Smoking**					< 0.001
Yes	48,996 (10.5%)	371 (16.7%)	620 (13.7%)	48,005 (10.5%)	
No	416,997 (89.5%)	1844 (83.3%)	3893 (86.3%)	411,260 (89.5%)	
**Alcohol**					< 0.001
Yes	428,357 (92.0%)	1710 (77.4%)	3894 (86.5%)	422,753 (92.1%)	
No	37,473 (8.0%)	500 (22.6%)	610 (13.5%)	36,363 (7.9%)	
**Loneliness**					< 0.001
Yes	84,529 (18.5%)	673 (31.3%)	1069 (24.2%)	82,787 (18.3%)	
No	373,594 (81.5%)	1480 (68.7%)	3343 (75.8%)	368,771 (81.7%)	
**Serum IGF-1**,** nmol/L**	21.40 ± 5.70	19.57 ± 6.43	20.38 ± 5.91	21.42 ± 5.69	< 0.001
**Visuospatial memory**	4.15 ± 3.39	4.48 ± 3.93	4.51 ± 3.81	4.15 ± 3.38	< 0.001
**Prospective memory**					< 0.001
Good	29,348 (18.5%)	234 (28.5%)	363 (23.1%)	28,751 (18.4%)	
Medium	121,598 (76.5%)	502 (61.1%)	1090 (69.4%)	120,006 (76.7%)	
Poor	7997 (5.0%)	85 (10.4%)	118 (7.5%)	7794 (5.0%)	
**Numeric memory**	6.48 ± 1.83	5.70 ± 2.31	6.10 ± 2.07	6.49 ± 1.82	< 0.001
**Reaction time**	559.16 ± 117.65	619.27 ± 172.87	592.58 ± 142.66	558.55 ± 116.93	< 0.001
**Fluid intelligence**	5.99 ± 2.16	5.16 ± 2.25	5.61 ± 2.19	5.99 ± 2.16	< 0.001

Data are presented as number (%) for categorical variables and mean ± standard deviation for continuous variables. Overall differences across the three groups were evaluated using the χ² test for categorical variables, one-way ANOVA for continuous variables with approximately normal distributions and homogeneity of variance, or the Kruskal–Wallis test for non-normally distributed variables. The *P*-values shown represent overall group comparisons. Post hoc pairwise comparisons were conducted only when the overall test was significant and are reported in STable 1 with multiplicity correction

Post hoc pairwise comparisons with multiplicity correction are provided in STable 1. Participants in the incident epilepsy group were older than both those with epilepsy at baseline and those who remained epilepsy-free throughout follow-up (both adjusted *P* < 0.001), whereas age did not differ significantly between the baseline epilepsy group and the epilepsy-free group (adjusted *P* = 1.000). Compared with participants who remained epilepsy-free throughout follow-up, both the baseline epilepsy group and the incident epilepsy group had higher BMI, higher IMD, higher proportions of smoking and loneliness, a lower proportion reporting alcohol use, fewer working hours per week, lower serum IGF-1 levels, and less favorable performance across several cognitive domains. No significant pairwise difference was observed between the baseline epilepsy group and the incident epilepsy group for BMI, numeric memory, visuospatial memory, or working hours per week after multiplicity correction.

### Lower serum IGF-1 levels and less favorable cognitive performance across epilepsy-status groups

Serum IGF-1 levels differed significantly across the three groups (*P* overall < 0.001). Participants with epilepsy at baseline had the lowest mean serum IGF-1 level (19.57 ± 6.43 nmol/L); the incident epilepsy group had intermediate levels (20.38 ± 5.91 nmol/L); and participants who remained epilepsy-free throughout follow-up had the highest levels (21.42 ± 5.69 nmol/L). Post hoc comparisons confirmed that all three pairwise differences remained significant after multiplicity correction (all adjusted *P* < 0.001).

Cognitive performance also differed across epilepsy-status groups. Significant overall group differences were observed for visuospatial memory, prospective memory, numeric memory, reaction time, and fluid intelligence (all *P* overall < 0.001). In post hoc analyses, both the baseline epilepsy group and the incident epilepsy group showed less favorable performance than participants who remained epilepsy-free throughout follow-up in several domains, particularly in reaction time, numeric memory, and fluid intelligence. Reaction time was longest in the baseline epilepsy group, intermediate in the incident epilepsy group, and shortest in the epilepsy-free group; all pairwise comparisons were significant after multiplicity correction (all adjusted *P* < 0.001). Fluid intelligence was lowest in the baseline epilepsy group, intermediate in the incident epilepsy group, and highest in the epilepsy-free group, with all pairwise comparisons remaining significant after multiplicity correction (all adjusted *P* < 0.001). Together, these findings show that both the baseline epilepsy group and the incident epilepsy group had lower serum IGF-1 levels and less favorable cognitive profiles than participants who remained epilepsy-free throughout follow-up.

### Serum IGF-1 concentrations and risk of epilepsy

**Fig. 1:**
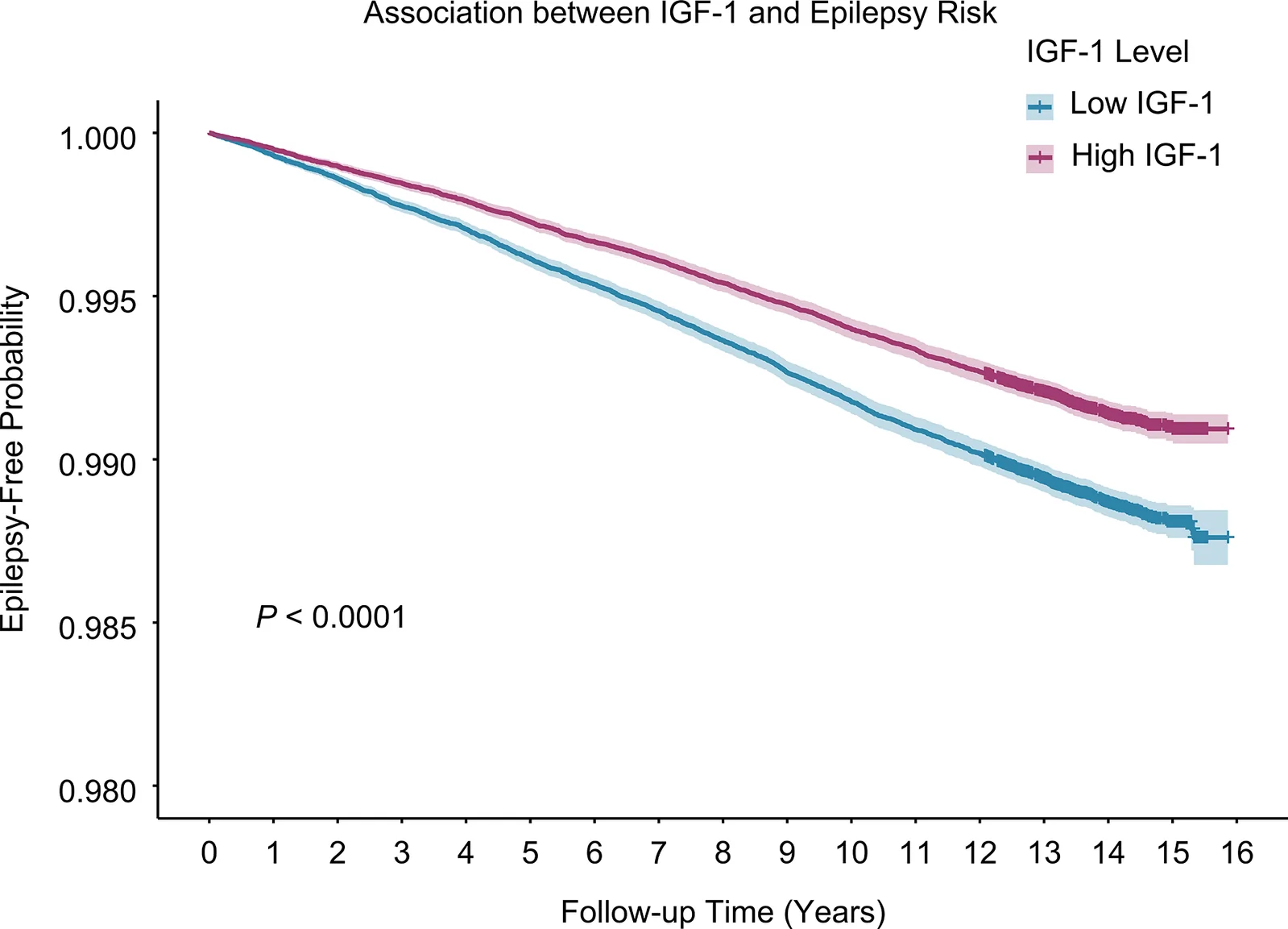
Kaplan–Meier analysis of epilepsy-free survival by baseline serum IGF-1 levels. The curve compares participants stratified according to serum IGF-1 concentrations above (High IGF-1) or below (Low IGF-1) the median value. Log-rank testing demonstrated significantly longer epilepsy-free survival in the High-IGF-1 group (*P* < 0.0001), indicating an increased risk of epilepsy development among individuals with lower baseline serum IGF-1 levels. The numbers of participants at risk in the High-IGF-1 and Low-IGF-1 groups were 232,316 and 232,316 at baseline, 231,683 and 231,419 at 5 years, 230,921 and 230,403 at 10 years, and 14,551 and 15,589 at 15 years, respectively

Among participants free of epilepsy at baseline, 4,532 developed epilepsy during follow-up. Kaplan–Meier analysis based on the median baseline serum IGF-1 level showed significantly better epilepsy-free survival in participants with higher IGF-1 levels than in those with lower levels (log-rank *P* < 0.0001; Fig. 1), indicating a higher long-term risk of epilepsy among individuals with lower baseline serum IGF-1 concentrations.

**Fig. 2:**
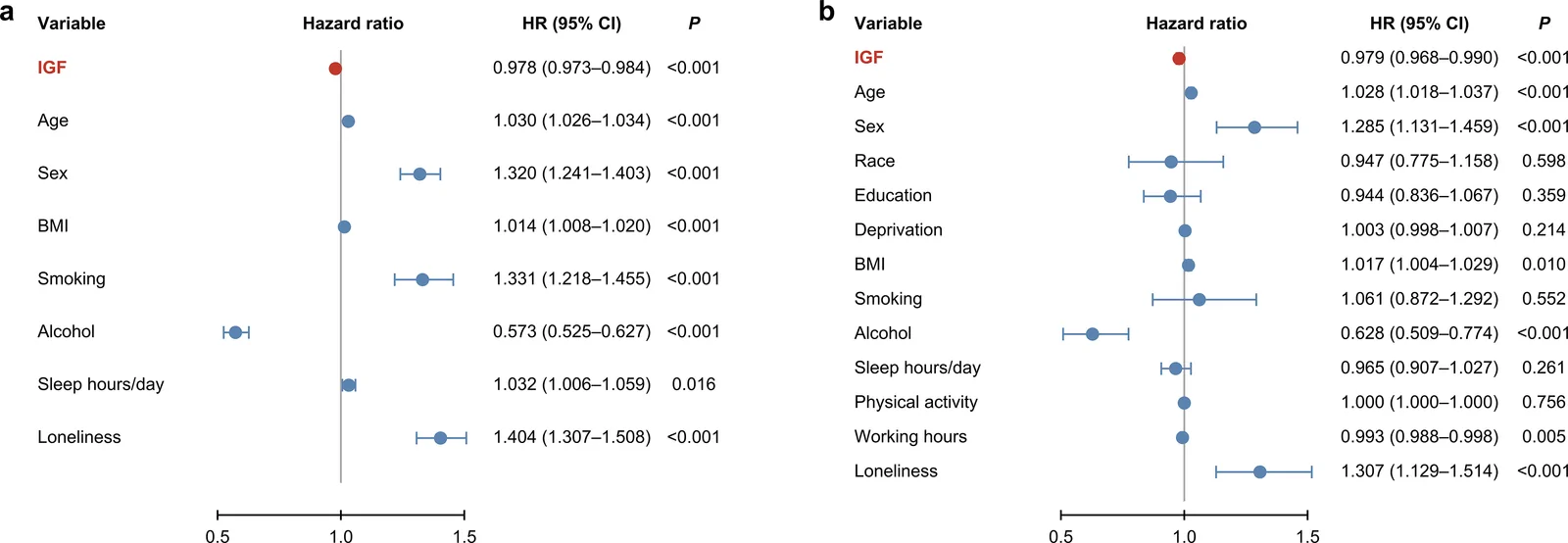
Prespecified Cox proportional hazards models for incident epilepsy. **a** Primary model, adjusted for age, sex, BMI, smoking, alcohol use, sleep hours per day, and loneliness. **b** Sensitivity model, additionally adjusted for ethnicity, education, deprivation, physical activity, and working hours in a reduced complete-case sample. Dots represent hazard ratios, horizontal lines represent 95% confidence intervals, and the vertical dashed line indicates HR = 1.0

To evaluate this association more rigorously, we constructed prespecified Cox proportional hazards models based on available covariates rather than selecting variables according to univariable significance. Across both prespecified models, higher serum IGF-1 levels remained consistently associated with a lower risk of incident epilepsy (STable 2). In the primary model, adjusted for age, sex, BMI, smoking, alcohol use, sleep hours per day, and loneliness, each 1 nmol/L increase in serum IGF-1 was associated with a 2.2% lower hazard of incident epilepsy (HR = 0.978, 95% CI 0.973–0.984, *P* < 0.001; Fig. 2a). In the sensitivity model, which additionally included ethnicity, education, deprivation, physical activity, and working hours in a reduced complete-case sample, the inverse association remained materially unchanged (HR = 0.979, 95% CI 0.968–0.990, *P* < 0.001; Fig. 2b), supporting the robustness of the main finding despite the smaller analytic sample.

To improve the clinical interpretability of the effect size, we additionally examined serum IGF-1 as both a standardized continuous variable and a categorical exposure defined by quartiles (STable 5). In the primary model, each 1 SD increase in serum IGF-1 was associated with a lower hazard of incident epilepsy (HR = 0.882, 95% CI 0.854–0.912, *P* < 0.001). When participants were categorized into quartiles, those in Q2, Q3, and Q4 had lower risks of incident epilepsy than those in Q1, with hazard ratios of 0.783 (95% CI 0.722–0.849), 0.737 (95% CI 0.677–0.802), and 0.755 (95% CI 0.692–0.823), respectively (all *P* < 0.001). Similar results were observed in the sensitivity model, in which the corresponding hazard ratios were 0.733 (95% CI 0.618–0.869, *P* < 0.001), 0.744 (95% CI 0.629–0.880, *P* < 0.001), and 0.760 (95% CI 0.642–0.901, *P* = 0.002), respectively. A significant trend across quartiles was observed in both the primary and sensitivity models (both *P* for trend ≤ 0.004).

Absolute risk estimates were also consistent with these relative risk reductions. The incidence rates per 1,000 person-years were 0.937 in Q1, 0.687 in Q2, 0.621 in Q3, and 0.604 in Q4. Similarly, the estimated 15-year cumulative incidence of epilepsy was highest in Q1 (1.382%) and lower in Q2 (1.000%), Q3 (0.904%), and Q4 (0.896%) (STable 5; SFigure 2). Notably, the quartile-based results did not show a strictly monotonic decline from Q3 to Q4, suggesting a plateau in the inverse association at higher IGF-1 concentrations.

To further examine the possibility of reverse causation, we performed lagged Cox analyses excluding epilepsy events occurring within the first 2 and 5 years of follow-up. The inverse association between serum IGF-1 and incident epilepsy remained directionally consistent and statistically significant in both the primary and sensitivity models (SFigure 1; STable 4). In the primary model, the hazard ratios per 1 nmol/L increase in serum IGF-1 were 0.980 (95% CI 0.974–0.986, *P* < 0.001) after excluding events within the first 2 years and 0.985 (95% CI 0.978–0.992, *P* < 0.001) after excluding events within the first 5 years. In the sensitivity model, the corresponding hazard ratios were 0.979 (95% CI 0.968–0.991, *P* < 0.001) and 0.981 (95% CI 0.967–0.994, *P* = 0.005), respectively. These findings suggest that the observed inverse association was not solely driven by events occurring shortly after baseline.

Because the inverse association between alcohol use and incident epilepsy in the primary analysis was potentially counterintuitive, we performed sensitivity analyses using a more detailed alcohol-frequency coding scheme based on the available baseline UK Biobank categories (STable 6). Replacing the binary alcohol variable with refined alcohol categories did not materially alter the association between serum IGF-1 and incident epilepsy. In the primary model, the hazard ratio for serum IGF-1 remained 0.978 (95% CI 0.973–0.984, *P* < 0.001), and in the sensitivity model it remained 0.979 (95% CI 0.968–0.990, *P* < 0.001). Compared with never drinkers, occasional, regular weekly, and daily/almost daily drinkers all showed lower hazards of incident epilepsy. In the primary model, the corresponding hazard ratios were 0.660 (95% CI 0.596–0.731, *P* < 0.001), 0.540 (95% CI 0.491–0.594, *P* < 0.001), and 0.545 (95% CI 0.489–0.607, *P* < 0.001), respectively. Similar results were observed in the sensitivity model, with hazard ratios of 0.728 (95% CI 0.576–0.921, *P* = 0.008), 0.591 (95% CI 0.475–0.736, *P* < 0.001), and 0.606 (95% CI 0.476–0.771, *P* < 0.001), respectively. Notably, the inverse association did not become progressively stronger at the highest drinking frequency. In an additional sensitivity analysis excluding participants who reported drinking daily or almost daily, the inverse association between serum IGF-1 and incident epilepsy remained materially unchanged in both the primary and sensitivity models (STable 6).

Prespecified subgroup analyses were further conducted by age and sex (STable 8; SFigure 4). In the primary model, the inverse association between serum IGF-1 and incident epilepsy was observed in both age strata, but was stronger among participants aged < 60 years (HR = 0.970, 95% CI 0.962–0.978, *P* < 0.001) than among those aged ≥ 60 years (HR = 0.985, 95% CI 0.978–0.993, *P* < 0.001), with evidence of interaction (*P* for interaction = 0.003). However, this age-related effect modification was not replicated in the sensitivity model (*P* for interaction = 0.663). By contrast, the inverse association between serum IGF-1 and incident epilepsy was broadly consistent across sex strata in both the primary and sensitivity models, with no significant evidence of interaction (*P* for interaction = 0.279 and 0.300, respectively). These findings suggest that the association was generally stable across sex strata, whereas evidence for age-related effect modification was inconsistent across models and should therefore be interpreted cautiously.

**Fig. 3:**
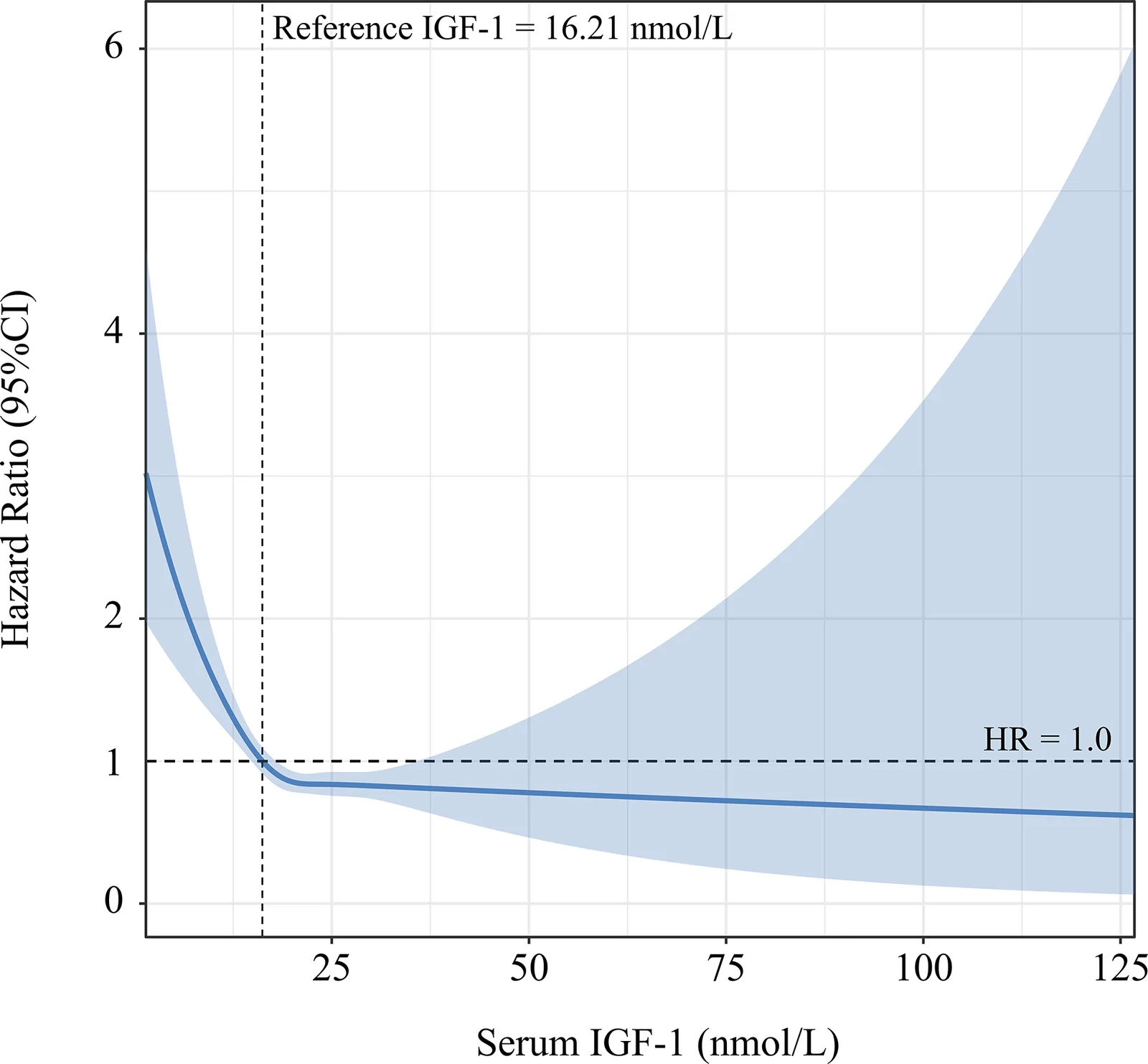
Nonlinear association between serum IGF-1 concentration and incident epilepsy using restricted cubic splines. Restricted cubic spline analysis was conducted within the prespecified Cox modeling framework. Five knots were placed at the 5th, 35th, 50th, 65th, and 95th percentiles of the serum IGF-1 distribution. A significant L-shaped nonlinear association was observed (*P* for nonlinearity < 0.001), with a steeper increase in epilepsy risk at lower IGF-1 concentrations and an apparent plateau at higher concentrations. The solid curve represents the hazard ratio, and the shaded area represents the 95% confidence interval

Given that serum IGF-1 remained associated with incident epilepsy across both prespecified models, lagged sensitivity analyses, alcohol-frequency sensitivity analyses, and subgroup analyses, we further explored the dose-response relationship using restricted cubic splines (Fig. 3). A significant L-shaped nonlinear association was observed, with a steeper increase in epilepsy risk at lower IGF-1 levels and a plateau at higher concentrations (*P* for nonlinearity < 0.001). The apparent inflection point was observed near 20 nmol/L, supporting the observation that epilepsy risk rose more steeply at lower serum IGF-1 concentrations.

### Serum IGF-1 and cognitive performance in participants with epilepsy at baseline

We further examined whether serum IGF-1 was related to cognitive performance in participants with epilepsy at baseline. In the fully adjusted model (Table 2), higher serum IGF-1 was nominally associated with faster reaction time (β = – 1.588, 95% CI – 2.997 to –0.180, *P* = 0.027) and a more favorable prospective memory category (OR = 1.033, 95% CI 1.003 to 1.063, *P* = 0.031). No statistically significant associations were observed for visuospatial memory (β = – 0.021, 95% CI – 0.053 to 0.012, *P* = 0.209), numeric memory (β = 0.054, 95% CI – 0.007 to 0.114, *P* = 0.084), or fluid intelligence (β = 0.018, 95% CI – 0.013 to 0.050, *P* = 0.257).

**Table 2:** Fully adjusted associations of serum IGF-1 with cognition at baseline

Cognitive domain	Model type	Effect estimate	95% CI	Raw *P* value
Reaction time	Linear regression	β = – 1.588	– 2.997 to –0.180	0.027
Visuospatial memory	Linear regression	β = – 0.021	– 0.053 to 0.012	0.209
Numeric memory	Linear regression	β = 0.054	– 0.007 to 0.114	0.084
Fluid intelligence	Linear regression	β = 0.018	– 0.013 to 0.050	0.257
Prospective memory	Ordinal logistic regression	OR = 1.033	1.003 to 1.063	0.031

Fully adjusted associations between baseline serum IGF-1 and cognitive performance in participants with epilepsy at baseline. Linear regression was used for reaction time, visuospatial memory, numeric memory, and fluid intelligence; ordinal logistic regression was used for prospective memory. Effect estimates are presented as β coefficients for continuous outcomes and odds ratios (ORs) for prospective memory, per 1 nmol/L increase in serum IGF-1

Because five cognitive domains were analyzed, we additionally applied the Benjamini–Hochberg false discovery rate correction to the fully adjusted model results (STable 7). After FDR correction, none of the associations remained statistically significant, including reaction time and prospective memory (both *q* = 0.078). These findings suggest that the cross-sectional associations between serum IGF-1 and cognitive performance in participants with epilepsy at baseline should be interpreted cautiously.

### Exploratory longitudinal analyses of serum IGF-1 and cognitive performance

**Fig. 4:**
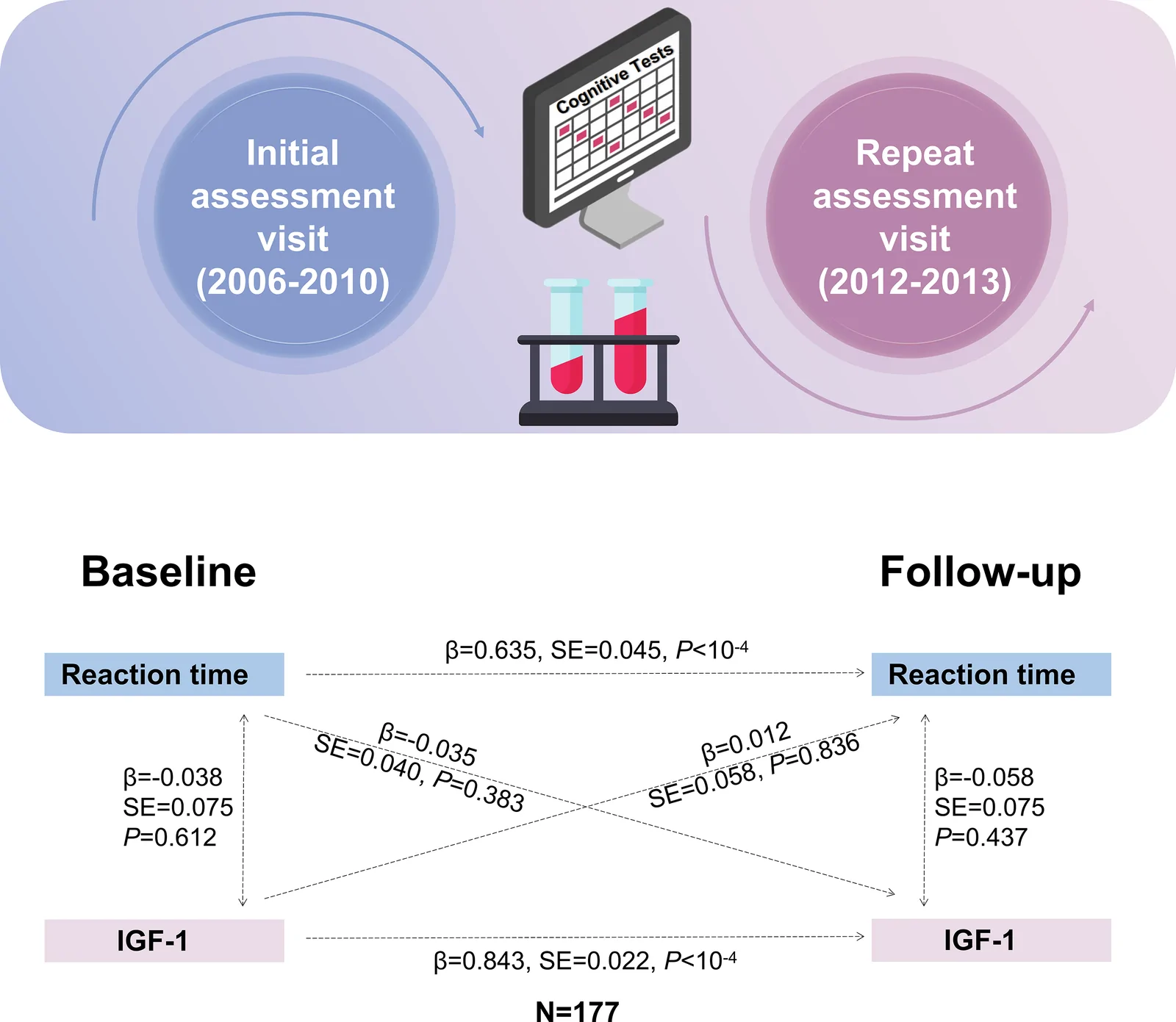
Exploratory cross-lagged model of IGF-1 and reaction time. After adjustment for covariates, baseline serum IGF-1 did not significantly predict follow-up reaction time (β = 0.012, *P* = 0.836), and baseline reaction time did not significantly predict follow-up serum IGF-1 (β = – 0.035, *P* = 0.383). Given the limited sample size, this analysis is exploratory and should not be interpreted as confirmatory evidence regarding cognitive trajectories in epilepsy

To explore the temporal relationship between serum IGF-1 and cognitive performance, we conducted longitudinal analyses using repeated cognitive data. In the subgroup of participants with epilepsy at baseline, only 177 individuals had complete longitudinal data for both serum IGF-1 and reaction time. In this exploratory cross-lagged panel model, baseline serum IGF-1 did not significantly predict follow-up reaction time (β = 0.012, *P* = 0.836), and baseline reaction time did not significantly predict follow-up serum IGF-1 (β = – 0.035, *P* = 0.383) (Fig. 4). Given the limited sample size, these subgroup analyses were considered exploratory and were not interpreted as providing confirmatory evidence regarding cognitive trajectories in epilepsy.

Because longitudinal data were limited in the epilepsy subgroup, we additionally examined the association between baseline serum IGF-1 and subsequent cognitive performance in the full cohort (SFigure 3, panels a–d). After adjustment for confounding variables, higher baseline serum IGF-1 was associated with better subsequent performance in reaction time (β = – 0.049, *P* < 0.001), visuospatial memory (β = – 0.030, *P* < 0.001), and fluid intelligence (β = 0.032, *P* = 0.010), whereas no significant association was observed for prospective memory (β = 0.014, *P* = 0.379). Baseline cognitive performance did not significantly predict follow-up serum IGF-1 in any of the available domains. Analyses involving numeric memory were not feasible because of insufficient longitudinal data. These findings from the full cohort should be interpreted as hypothesis-generating and not as epilepsy-specific longitudinal evidence.

Overall, the longitudinal analyses provide limited evidence for a temporal association between serum IGF-1 and cognitive performance in participants with epilepsy at baseline, while the full-cohort findings suggest that lower baseline IGF-1 may be associated with less favorable later cognitive performance at the population level. However, these observations require confirmation in larger, epilepsy-specific longitudinal cohorts.

## Discussion

IGF-1 is increasingly recognized as a biologically relevant component of brain health, given its roles in neurodevelopment, neuronal survival, synaptic plasticity, glial function, and neuroimmune regulation [21]. In parallel, contemporary epilepsy research has emphasized that the clinical burden of epilepsy extends beyond seizure recurrence to include cognitive dysfunction, reduced quality of life, and complex interactions with aging, treatment exposure, and comorbidity burden [7, 41–43]. Against this background, the present study examined whether circulating IGF-1 was associated with incident epilepsy and cognitive measures in a large population-based cohort.

Our main finding was that lower baseline serum IGF-1 concentrations were consistently associated with a higher risk of incident epilepsy. This association remained stable across prespecified Cox models and multiple sensitivity analyses, including quartile-based analyses, lagged Cox analyses, alternative alcohol coding, and subgroup analyses. In the primary model, each 1 nmol/L increase in serum IGF-1 was associated with a 2.2% lower hazard of incident epilepsy, and a comparable estimate was observed in the more extensively adjusted sensitivity model. When effect size was expressed in more clinically interpretable ways, each 1 SD increase in IGF-1 was associated with an approximately 11–12% lower hazard of incident epilepsy, and participants in higher IGF-1 quartiles had consistently lower risks than those in the lowest quartile. Absolute risk estimates were directionally concordant, with lower incidence rates and lower estimated 15-year cumulative incidence in the higher quartiles. Taken together, these findings support a robust population-level inverse association between circulating IGF-1 and incident epilepsy.

The shape of the association is also noteworthy. The quartile-based analyses did not show a strictly monotonic decline in risk from Q3 to Q4, and the restricted cubic spline analysis demonstrated an L-shaped relationship, with a steeper increase in epilepsy risk at lower IGF-1 concentrations and an apparent plateau at higher concentrations. This pattern argues against a simplistic linear interpretation in which progressively higher IGF-1 is associated with proportionally lower epilepsy risk across the entire range. Instead, it suggests that lower circulating IGF-1 may mark a biological state in which epilepsy susceptibility rises more sharply, whereas at higher concentrations the apparent protective association plateaus. Importantly, the apparent inflection point near 20 nmol/L should not be interpreted as a clinically validated threshold for intervention, but rather as a feature of the observed exposure–response pattern in this cohort.

We also addressed the possibility that the observed association reflected early preclinical disease or reverse causation rather than a more durable epidemiological relationship. In lagged Cox analyses excluding epilepsy events occurring within the first 2 and 5 years of follow-up, the inverse association between serum IGF-1 and incident epilepsy remained directionally consistent and statistically significant in both the primary and sensitivity models. These results reduce the concern that the main finding is explained solely by short-term reverse causation or preclinical disease immediately preceding diagnosis. However, they do not eliminate this possibility entirely, and the present study remains observational. Similarly, prespecified subgroup analyses suggested that the inverse association was broadly consistent across sex strata. Although an age interaction was observed in the primary model, it was not reproduced in the sensitivity model and should therefore be interpreted cautiously rather than as firm evidence of effect modification.

From a biological perspective, our findings are plausible, but they should not be overinterpreted mechanistically. IGF-1 signaling has long been implicated in neuronal differentiation, synaptic maintenance, and resistance to cellular stress, and more recent work has highlighted its relevance to human brain development, including the IGF1-dependent microglial regulation of prenatal GABAergic neurogenesis [22]. Such observations are relevant to epilepsy, in which inhibitory–excitatory imbalance, network maturation, and neuroinflammatory signaling are central themes. At the same time, the current study does not establish that reduced circulating IGF-1 is a direct cause of epileptogenesis, nor does it identify the specific pathways linking IGF-1 to incident epilepsy in humans. This distinction is important, particularly because circulating IGF-1 is itself influenced by age, metabolic state, endocrine physiology, and systemic illness [19]. Our results are therefore better regarded as epidemiological evidence supporting IGF-1 as a candidate biomarker and biologically relevant pathway rather than proof of a direct mechanistic or therapeutic effect.

The alcohol findings also warrant a restrained interpretation [43–45]. In the primary analysis, alcohol use appeared to be associated with a lower incidence of epilepsy, which is potentially counterintuitive from a clinical perspective. Sensitivity analyses using a more detailed baseline drinking-frequency variable showed that this pattern persisted beyond a simple binary yes/no alcohol coding, but it did not become progressively stronger at the highest drinking frequency. This argues against a straightforward protective effect of alcohol. More likely, the observed pattern reflects residual confounding, healthy-user effects, or limitations of the available dataset, particularly the inability to distinguish former drinkers from lifelong abstainers and the lack of more precise quantification of drinking volume and pattern.

The cognitive findings were substantially more limited than the epilepsy-risk analyses and should be interpreted accordingly. In the baseline epilepsy sample, higher IGF-1 was nominally associated with faster reaction time and better prospective memory in fully adjusted cross-sectional models, but neither association remained statistically significant after false discovery rate correction across the five cognitive domains. No significant fully adjusted association was observed for visuospatial memory, numeric memory, or fluid intelligence. These findings indicate that, within the baseline epilepsy subgroup, the evidence for a robust cross-sectional relationship between serum IGF-1 and cognition is modest. The direction of the nominal associations is biologically plausible and aligns with the broader literature linking IGF-1 to cognitive health [20, 30, 44, 45], but after accounting for multiple testing, the present data do not support strong domain-specific conclusions.

The longitudinal cognitive results require even greater caution. In the epilepsy subgroup, the cross-lagged panel analysis included only 177 participants with complete longitudinal data for both serum IGF-1 and reaction time and did not identify significant bidirectional associations. These analyses should therefore be regarded as exploratory rather than confirmatory and do not support strong inferences about cognitive trajectories in people with epilepsy. By contrast, in the full cohort, higher baseline IGF-1 was associated with better subsequent performance in several cognitive domains. However, these full-cohort analyses were not epilepsy-specific and should be interpreted as population-level longitudinal observations rather than evidence that IGF-1 influences cognitive decline in epilepsy. This distinction is particularly important in light of recent work showing that cognition in epilepsy is shaped by multiple interacting determinants, including seizure activity, treatment exposure, age at onset, and underlying disease substrate [7, 42, 46].

This study has several strengths. The use of the UK Biobank provided a large, prospective, population-based cohort with long follow-up, allowing the association between baseline serum IGF-1 and incident epilepsy to be examined with substantially greater precision than is usually possible in clinically recruited epilepsy samples. The standardized measurement of serum IGF-1 and the availability of multidomain cognitive assessments further strengthened the dataset. In addition, the analytic framework improved the robustness of interpretation by using prespecified Cox models, clinically interpretable effect-size displays, lagged analyses, alternative alcohol coding, and subgroup analyses. These features do not eliminate the limitations of the study, but they do reduce the likelihood that the main association is a simple artifact of model specification.

Several limitations remain important. First, epilepsy in this cohort was ascertained at the population level rather than through detailed syndrome-specific clinical phenotyping, so we could not account for epilepsy etiology, seizure frequency, EEG findings, imaging-defined substrate, or antiseizure medication exposure [13, 47]. This is particularly relevant in neurology, because both epilepsy subtype and treatment can influence cognition and potentially interact with endocrine or metabolic pathways. Second, the primary and sensitivity models were built from available sociodemographic and lifestyle variables but did not include detailed vascular, metabolic, psychiatric, neurological, or medication-related covariates such as diabetes treatment, antihypertensive therapy, or antiseizure medications [48, 49]. Residual confounding therefore remains possible. Third, serum IGF-1 was measured at a single baseline time point, which may not adequately reflect long-term exposure to the GH/IGF axis or the capture within-person trajectory over time. Fourth, cognition was not assessed synchronously and repeatedly across all participants in a way that would allow a definitive epilepsy-specific temporal model, and the longitudinal epilepsy subgroup was small. Finally, because the cohort was drawn predominantly from middle-aged adults of European ancestry, generalizability to younger populations, very old adults, and more diverse populations remains uncertain.

Overall, the revised analyses support the interpretation of serum IGF-1 as a robust epidemiological correlate of incident epilepsy rather than as a direct causal factor or established therapeutic target. The consistency of the association across prespecified adjustment strategies, alternative effect-size presentations, lagged analyses, alcohol-frequency sensitivity analyses, and selected subgroup analyses strengthens confidence that the result is not merely statistical noise. At the same time, the more limited and largely non-confirmatory cognitive findings underscore the need to separate relatively strong evidence for an epilepsy-risk association from much weaker evidence for epilepsy-specific cognitive effects. Future studies with richer clinical phenotyping, repeated biomarker measurements, detailed medication data, and adequately powered epilepsy-specific longitudinal designs will be required to determine whether IGF-1 improves risk stratification, whether it plays a causal role in epileptogenesis, and whether modulation of the IGF-1 axis has any meaningful clinical relevance in epilepsy.

## Conclusions

In this large population-based cohort, lower serum IGF-1 concentrations were independently associated with a higher risk of incident epilepsy. The observed association was nonlinear and remained broadly consistent across prespecified models and multiple sensitivity analyses, supporting IGF-1 as a robust epidemiological correlate of epilepsy risk at the population level. By contrast, evidence for cognitive associations was more limited: cross-sectional findings in participants with epilepsy at baseline were attenuated after multiple-testing correction, and epilepsy-specific longitudinal analyses were exploratory and underpowered. Taken together, these findings support further investigation of circulating IGF-1 as a candidate biomarker and biologically relevant pathway rather than as evidence of a causal mechanism or established therapeutic target. Future studies incorporating richer clinical phenotyping, repeated biomarker measurements, and adequately powered longitudinal or interventional designs will be required to clarify whether IGF-1 has clinical utility in epilepsy risk stratification or cognitive outcome prediction.

## Supplementary Information

Below is the link to the electronic supplementary material.

Supplementary Material 1

## Data Availability

All data from the UK Biobank are available to researchers through application via the Access.

## Abbreviations

BDNF: Brain-Derived Neurotrophic Factor
BMI: Body Mass Index
CI: Confidence Interval
CGRP: Calcitonin Gene-Related Peptide
EEG: Electroencephalogram
EQA: External Quality Assurance
HR: Hazard Ratio
ICD-10: International Classification of Diseases, Tenth Revision
IGF-1: Insulin-like Growth Factor 1
IGF-1R: Insulin-like Growth Factor 1 Receptor
IMD: Index of Multiple Deprivation
MAPK/ERK: Mitogen-Activated Protein Kinase / Extracellular Signal-Regulated Kinase
NHS: National Health Service
PI3K/Akt: Phosphoinositide 3-Kinase / Protein Kinase B
RCS: Restricted Cubic Splines
