# Supplementary material for: Serum insulin-like growth factor-1 and its association with epilepsy risk and cognitive function
Source: Acta Epileptol. 2026 Oct 1;8:36. doi: 10.1186/s42494-026-00274-2 (PMC13628725; doi:10.1186/s42494-026-00274-2)
Supplement: Supplementary file 1 [file 42494_2026_274_MOESM1_ESM.docx]

**Supplemental Online Content**

**SFigure 1. Association of serum IGF-1 with incident epilepsy in the main and lagged Cox analyses.**

**SFigure 2. Kaplan–Meier curves for incident epilepsy according to quartiles of serum IGF-1.**

**SFigure 3. Longitudinal association between serum IGF-1 levels and performance on different cognitive tests in the entire cohort of participants (N = 3,710–16,350).**

**SFigure 4. Subgroup and interaction analyses for the association between serum IGF-1 and incident epilepsy.**

**STable 1. Post hoc pairwise comparisons of baseline characteristics across epilepsy-status groups.**

**STable 2. Prespecified Cox proportional hazards models for incident epilepsy.**

**STable 3. Proportional hazards assumption, missing data, and model notes for the Cox analyses.**

**STable 4. Lagged Cox analyses excluding epilepsy events occurring within the first 2 and 5 years of follow-up.**

**STable 5. Association of serum IGF-1 quartiles and per 1 SD increase with incident epilepsy, including event counts, person-years, incidence rates, and 15-year cumulative incidence.**

**STable 6. Alternative alcohol coding sensitivity analysis for the association between serum IGF-1 and incident epilepsy.**

**STable 7. FDR-adjusted results for the associations between serum IGF-1 and cognitive performance across domains.**

**STable 8. Subgroup and interaction analyses for the association between serum IGF-1 and incident epilepsy.**

**SFigure 1. Association of serum IGF-1 with incident epilepsy in the main and lagged Cox analyses.**

Forest plot showing the hazard ratios and 95% confidence intervals for serum IGF-1 in the main analysis and in lagged Cox analyses excluding epilepsy events occurring within the first 2 and 5 years of follow-up. Results are shown for the primary model, adjusted for age, sex, BMI, smoking, alcohol use, sleep hours per day, and loneliness, and for the sensitivity model, additionally adjusted for ethnicity, education, deprivation, physical activity, and working hours in a reduced complete-case sample. The vertical dashed line indicates HR = 1.0.

**SFigure 2. Kaplan–Meier curves for incident epilepsy according to quartiles of serum IGF-1.**

Kaplan–Meier curves showing epilepsy-free survival according to quartiles of baseline serum IGF-1 concentration (Q1–Q4). Participants in higher quartiles showed better epilepsy-free survival during follow-up. The estimated 15-year cumulative incidence of epilepsy was 1.382% in Q1, 1.000% in Q2, 0.904% in Q3, and 0.896% in Q4, consistent with the relative risk estimates from the quartile-based Cox models shown in STable 5. Quartiles were defined according to the baseline serum IGF-1 distribution in the full cohort.

**SFigure 3.** **Longitudinal association between serum IGF-1 levels and performance on different cognitive tests in the entire cohort of participants (N = 3,710–16,350).**

(a) For visuospatial memory assessment, higher baseline IGF-1 levels predicted better follow-up performance (β=-0.030, *P*<10^-4^), whereas baseline visuospatial memory did not predict follow-up IGF-1 levels (β=-0.002, *P*=0.696).

(b) For prospective memory assessment, neither baseline IGF-1 levels predicted follow-up performance (β=0.014, *P*=0.379) nor did baseline performance predict follow-up IGF-1 levels (β=-0.007, *P*=0.470).

(c) For reaction time assessment, elevated baseline IGF-1 predicted shorter follow-up reaction times (β=-0.049, *P*<10^-4^), while baseline reaction time showed no association with follow-up IGF-1 levels (β=-0.006, *P*=0.242).

(d) For fluid intelligence scores assessment, higher baseline IGF-1 predicted improved follow-up performance (β=0.032, *P*=0.010), but baseline scores did not predict follow-up IGF-1 levels (β=0.018, *P*=0.072).

**SFigure 4. Subgroup and interaction analyses for the association between serum IGF-1 and incident epilepsy.**

Forest plot showing subgroup-specific hazard ratios and 95% confidence intervals for serum IGF-1 in relation to incident epilepsy, expressed per 1 nmol/L increase in serum IGF-1. Results are shown for age-stratified analyses (<60 years and ≥60 years) and sex-stratified analyses (female and male) under both the primary model and the sensitivity model. The primary model was adjusted for age, sex, BMI, smoking, alcohol use, sleep hours per day, and loneliness. The sensitivity model was additionally adjusted for ethnicity, education, deprivation, physical activity, and working hours in a reduced complete-case sample. For sex-stratified analyses, sex was omitted from the subgroup-specific models. The vertical dashed line indicates HR = 1.0.

**STable 1. Post hoc pairwise comparisons of baseline characteristics across epilepsy-status groups.**

| **Variable** | ***P* overall** | **Baseline epilepsy vs epilepsy-free** | **Incident epilepsy vs epilepsy-free** | **Baseline epilepsy vs Incident epilepsy** |
| --- | --- | --- | --- | --- |
| **Sex** | <0.001 | <0.001 | <0.001 | 1.000 |
| **Age, years** | <0.001 | 1.000 | <0.001 | <0.001 |
| **Education** | <0.001 | <0.001 | <0.001 | 0.062 |
| **BMI, kg/m²** | <0.001 | <0.001 | <0.001 | 1.000 |
| **IMD** | <0.001 | <0.001 | <0.001 | <0.001 |
| **Physical activity minutes per week** | <0.001 | <0.001 | 0.272 | 0.133 |
| **Working hours per week** | <0.001 | <0.001 | 0.002 | 0.101 |
| **Smoking** | <0.001 | <0.001 | <0.001 | 0.004 |
| **Alcohol** | <0.001 | <0.001 | <0.001 | <0.001 |
| **Loneliness** | <0.001 | <0.001 | <0.001 | <0.001 |
| **Serum IGF-1, nmol/L** | <0.001 | <0.001 | <0.001 | <0.001 |
| **Visuospatial memory** | <0.001 | 0.004 | <0.001 | 1.000 |
| **Prospective memory** | <0.001 | <0.001 | <0.001 | <0.001 |
| **Numeric memory** | <0.001 | <0.001 | <0.001 | 0.080 |
| **Reaction time** | <0.001 | <0.001 | <0.001 | <0.001 |
| **Fluid intelligence** | <0.001 | <0.001 | <0.001 | <0.001 |

Only variables with statistically significant overall group differences in Table 1 were included in this table. Overall comparisons across the three groups were performed using the χ² test for categorical variables, one-way ANOVA for continuous variables with approximately normal distributions and homogeneity of variance, or the Kruskal–Wallis test for non-normally distributed variables. Post hoc pairwise comparisons were conducted only when the overall test was significant. Tukey’s honestly significant difference test was used following ANOVA, Dunn’s test with Bonferroni correction was used following Kruskal–Wallis tests, and Bonferroni-corrected χ² tests were used for categorical variables. Values shown in this table are multiplicity-adjusted *P* values.

| **Model** | **Description** | **Analytic sample (n)** | **Events (n)** | **HR for serum IGF-1 (per 1 nmol/L)** | **95% CI** | ***P* value** |
| --- | --- | --- | --- | --- | --- | --- |
| Primary model | Adjusted for age, sex, BMI, smoking, alcohol use, sleep hours/day, and loneliness | 451,230 | 4,290 | 0.978 | 0.973–0.984 | <0.001 |
| Sensitivity model | Additionally adjusted for ethnicity, education, deprivation, physical activity, and working hours | 155,736 | 1,107 | 0.979 | 0.968–0.990 | <0.001 |

**STable 2. Prespecified Cox proportional hazards models for incident epilepsy.**

Primary model corresponds to the main multivariable model used in the manuscript. Sensitivity model corresponds to the more extensively adjusted reduced complete-case model. Incident epilepsy during follow-up was defined as status=1. The uploaded variable 'time' was used as the follow-up time variable. Cox proportional hazards models used the Breslow method for handling ties. Complete-case analysis was used for each model; therefore, the analytic sample size differs across models according to variable availability.

**STable 3. Proportional hazards assumption, missing data, and model notes for the Cox analyses.**

**Panel a. Proportional hazards assumption screening**

| **Model** | **Variable** | **Spearman rho** | **PH screening *P*** | **Interpretation** |
| --- | --- | --- | --- | --- |
| Primary model | Global screening (Fisher combined) |  | <0.001 | Screening only; interpret with caution in large samples |
| Primary model | Serum IGF-1 | 0.034 | 0.026 | Statistically detectable but very small time trend |
| Sensitivity model | Global screening (Fisher combined) |  | <0.001 | Screening only; interpret with caution in large samples |
| Sensitivity model | Serum IGF-1 | -0.000 | 0.989 | No evidence of PH violation |

PH screening was assessed using correlations between Schoenfeld residuals and log-transformed event time within the fitted Cox models. Because formal proportional hazards screening can be highly sensitive in very large samples, both the *P* value and the magnitude of rho should be interpreted.

**Panel b. Missingness of variables used in the Cox models**

| **Variable** | **Missing n** | **Missing %** |
| --- | --- | --- |
| Serum IGF-1 | 0 | 0.00 |
| Age | 0 | 0.00 |
| Sex | 0 | 0.00 |
| ethnicity | 34,823 | 7.49 |
| Education | 84,475 | 18.18 |
| Deprivation | 65,909 | 14.19 |
| BMI | 1,823 | 0.39 |
| Smoking | 854 | 0.18 |
| Alcohol use | 1,012 | 0.22 |
| Sleep hours/day | 3,468 | 0.75 |
| Physical activity | 89,113 | 19.18 |
| Working hours | 201,972 | 43.47 |
| Loneliness | 8,662 | 1.86 |

**Panel c. Model notes and analytic samples**

| **Model** | **Covariates** | **Analytic sample (n)** | **Events (n)** | **Missing-data approach** | **Time scale** |
| --- | --- | --- | --- | --- | --- |
| Primary model | Serum IGF-1, age, sex, BMI, smoking, alcohol use, sleep hours/day, loneliness | 451,230 | 4,290 | Complete-case analysis | Days from baseline to incident epilepsy or censoring |
| Sensitivity model | Serum IGF-1, age, sex, ethnicity, education, deprivation, BMI, smoking, alcohol use, sleep hours/day, physical activity, working hours, loneliness | 155,736 | 1,107 | Complete-case analysis | Days from baseline to incident epilepsy or censoring |

Missing data were handled using complete-case analysis. The primary model included serum IGF-1, age, sex, BMI, smoking, alcohol use, sleep hours/day, and loneliness. The sensitivity model additionally included ethnicity, education, deprivation, physical activity, and working hours.

**STable 4. Lagged Cox analyses excluding epilepsy events occurring within the first 2 and 5 years of follow-up.**

| **Analysis** | **Model** | **N after lag filter** | **Events after lag filter** | **Model N (complete cases)** | **Model events** | **IGF-1 HR (95% CI)** | ***P* value** |
| --- | --- | --- | --- | --- | --- | --- | --- |
| Main analysis | Primary model | 464,632 | 4,532 | 451,230 | 4,290 | 0.978 (0.973-0.984) | <0.001 |
| Main analysis | Sensitivity model | 464,632 | 4,532 | 155,736 | 1,107 | 0.979 (0.968-0.990) | <0.001 |
| 2-year lag | Primary model | 464,070 | 3,970 | 450,704 | 3,764 | 0.980 (0.974-0.986) | <0.001 |
| 2-year lag | Sensitivity model | 464,070 | 3,970 | 155,616 | 987 | 0.979 (0.968-0.991) | <0.001 |
| 5-year lag | Primary model | 463,102 | 3,002 | 449,793 | 2,853 | 0.985 (0.978-0.992) | <0.001 |
| 5-year lag | Sensitivity model | 463,102 | 3,002 | 155,406 | 777 | 0.981 (0.967-0.994) | 0.005 |

Participants with follow-up time less than or equal to the corresponding landmark time were excluded; follow-up time was redefined from the landmark onward. Primary model and sensitivity model used the same prespecified covariates as in the main Cox analyses.

**STable 5. Association of serum IGF-1 quartiles and per 1 SD increase with incident epilepsy, including event counts, person-years, incidence rates, and 15-year cumulative incidence.**

| **IGF-1 category** | **Median IGF-1 (nmol/L)** | **Range (nmol/L)** | **N** | **Events** | **Person-years** | **Incidence rate per 1,000 PY** | **15-year cumulative incidence** | **Primary model HR (95% CI)** | **Primary *P*** | **Sensitivity model HR (95% CI)** | **Sensitivity *P*** |
| --- | --- | --- | --- | --- | --- | --- | --- | --- | --- | --- | --- |
| Q1 (reference) | 15.02 | 1.45–17.53 | 116,171 | 1,490 | 1,589,599.3 | 0.937 | 1.382% | 1.00 (reference) | — | 1.00 (reference) | — |
| Q2 | 19.50 | 17.53–21.25 | 116,145 | 1,093 | 1,591,332.3 | 0.687 | 1.000% | 0.783 (0.722–0.849) | <0.001 | 0.733 (0.618–0.869) | <0.001 |
| Q3 | 22.93 | 21.25–24.82 | 116,159 | 989 | 1,591,431.0 | 0.621 | 0.904% | 0.737 (0.677–0.802) | <0.001 | 0.744 (0.629–0.880) | <0.001 |
| Q4 | 27.59 | 24.83–126.77 | 116,157 | 960 | 1,590,270.7 | 0.604 | 0.896% | 0.755 (0.692–0.823) | <0.001 | 0.760 (0.642–0.901) | 0.002 |
| Per 1 SD increase | 5.69 |  |  |  |  |  |  | 0.882 (0.854–0.912) | <0.001 | 0.887 (0.832–0.947) | <0.001 |
| *P* for trend |  |  |  |  |  |  |  | 0.976 (0.970–0.983) | <0.001 | 0.980 (0.967–0.994) | 0.004 |

Primary model was adjusted for age, sex, BMI, smoking, alcohol use, sleep hours/day, and loneliness. Sensitivity model was additionally adjusted for ethnicity, education, deprivation, physical activity, and working hours. Incidence rate is expressed per 1,000 person-years. The 15-year cumulative incidence was estimated using Kaplan–Meier methods. IGF-1 SD in the full cohort was 5.695 nmol/L.

**STable 6. Alternative alcohol coding sensitivity analysis for the association between serum IGF-1 and incident epilepsy.**

**Panel a. Refined alcohol-frequency coding in the Cox models**

| Variable comparison | Primary model HR (95% CI) | *P* value | Sensitivity model HR (95% CI) | *P* value |
| --- | --- | --- | --- | --- |
| Serum IGF-1 (per 1 nmol/L increase) | 0.978 (0.973–0.984) | <0.001 | 0.979 (0.968–0.990) | <0.001 |
| Occasional vs never | 0.660 (0.596–0.731) | <0.001 | 0.728 (0.576–0.921) | 0.008 |
| Regular weekly vs never | 0.540 (0.491–0.594) | <0.001 | 0.591 (0.475–0.736) | <0.001 |
| Daily/almost daily vs never | 0.545 (0.489–0.607) | <0.001 | 0.606 (0.476–0.771) | <0.001 |

**Panel b. Sensitivity analysis after excluding participants reporting daily or almost daily drinking**

| Variable / comparison | Primary model HR (95% CI) | *P* value | Sensitivity model HR (95% CI) | *P* value |
| --- | --- | --- | --- | --- |
| Serum IGF-1 (per 1 nmol/L increase) | 0.980 (0.974–0.987) | <0.001 | 0.979 (0.967–0.992) | 0.001 |
| Occasional vs never | 0.660 (0.595–0.731) | <0.001 | 0.727 (0.575–0.920) | 0.008 |
| Regular weekly vs never | 0.539 (0.490–0.593) | <0.001 | 0.589 (0.473–0.733) | <0.001 |

**Panel c. Descriptive distribution of baseline alcohol-frequency categories**

| Baseline alcohol frequency | N | Events | Person_years | Incidence rate per 1,000 PY |
| --- | --- | --- | --- | --- |
| Never | 36973 | 610 | 502994.1 | 1.213 |
| Special occasions only | 53264 | 628 | 726731.0 | 0.864 |
| One to three times a month | 51640 | 482 | 706815.6 | 0.682 |
| Once or twice a week | 119771 | 1056 | 1642742.6 | 0.643 |
| Three or four times a week | 107319 | 839 | 1473056.7 | 0.570 |
| Daily or almost daily | 94653 | 889 | 1296887.6 | 0.685 |

Alcohol frequency was re-coded using the available baseline UK Biobank categories. 'Prefer not to answer' and missing responses were treated as missing. Because information on former drinking was not available in the analytic dataset, participants were categorized as never, occasional (special occasions only or one to three times a month), regular weekly (once or twice a week or three or four times a week), or daily/almost daily. Both models used complete-case analysis.

Notes. In Panel a, the original binary alcohol variable was replaced with a refined alcohol-frequency variable based on the available baseline UK Biobank categories: never, occasional, regular weekly, and daily/almost daily drinking. In Panel b, participants who reported drinking daily or almost daily were excluded, and the Cox proportional hazards models were refitted. The primary model was adjusted for age, sex, BMI, smoking, alcohol, sleep hours per day, and loneliness. The sensitivity model was additionally adjusted for ethnicity, education, deprivation, physical activity, and working hours in a reduced complete-case sample. Hazard ratios are presented per 1 nmol/L increase in serum IGF-1. These analyses show that the inverse association between serum IGF-1 and incident epilepsy remained materially unchanged after alternative alcohol coding and after exclusion of daily/almost daily drinkers.

**STable 7. FDR-adjusted results for the associations between serum IGF-1 and cognitive performance across domains.**

| Cognitive domain | Effect estimate | 95% CI | Raw *P* value | FDR-adjusted q value | Interpretation |
| --- | --- | --- | --- | --- | --- |
| Reaction time | β = -1.588 | -2.997 to -0.180 | 0.027 | 0.078 | Not significant after FDR |
| Visuospatial memory | β = -0.021 | -0.053 to 0.012 | 0.209 | 0.257 | Not significant after FDR |
| Numeric memory | β = 0.054 | -0.007 to 0.114 | 0.084 | 0.140 | Not significant after FDR |
| Fluid intelligence | β = 0.018 | -0.013 to 0.050 | 0.257 | 0.257 | Not significant after FDR |
| Prospective memory | OR = 1.033 | 1.003 to 1.063 | 0.031 | 0.078 | Not significant after FDR |

Note. Benjamini–Hochberg FDR correction was applied across the five cognitive domains using the raw *P* values for the serum IGF-1 term from the fully adjusted model. None of the five associations remained significant after FDR correction. Reaction time and prospective memory were nominally significant only (q = 0.078).

**STable 8. Subgroup and interaction analyses for the association between serum IGF-1 and incident epilepsy.**

| Subgroup factor | Subgroup | Model | N | Events | HR (95% CI) | *P* | *P* for interaction |
| --- | --- | --- | --- | --- | --- | --- | --- |
| Age | <60 years | Primary model | 255335 | 1965 | 0.970 (0.962–0.978) | <0.001 | 0.003 |
| Age | <60 years | Sensitivity model | 127734 | 816 | 0.979 (0.966–0.991) | 0.001 | 0.663 |
| Age | ≥60 years | Primary model | 195895 | 2325 | 0.985 (0.978–0.993) | <0.001 |  |
| Age | ≥60 years | Sensitivity model | 28002 | 291 | 0.981 (0.959–1.004) | 0.100 |  |
| Sex | Female | Primary model | 244496 | 2073 | 0.980 (0.972–0.988) | <0.001 | 0.279 |
| Sex | Female | Sensitivity model | 79011 | 508 | 0.983 (0.967–0.999) | 0.038 | 0.300 |
| Sex | Male | Primary model | 206734 | 2217 | 0.976 (0.969–0.984) | <0.001 |  |
| Sex | Male | Sensitivity model | 76725 | 599 | 0.976 (0.960–0.991) | 0.002 |  |

Note. Primary model: adjusted for age, sex, BMI, smoking, alcohol use, sleep hours per day, and loneliness. Sensitivity model: additionally adjusted for ethnicity, education, deprivation, physical activity, and working hours in a reduced complete-case sample. For sex-stratified analyses, sex was omitted from the subgroup-specific models. *P* for interaction values were obtained by including product terms between serum IGF-1 and the subgroup variable in the corresponding Cox proportional hazards models.
